# Sesquiterpene Lactones Inhibit Advanced Oxidation Protein Product-Induced MCP-1 Expression in Podocytes via an IKK/NF-*κ*B-Dependent Mechanism

**DOI:** 10.1155/2015/934058

**Published:** 2015-01-12

**Authors:** Yan Zhao, Si-jia Chen, Jian-cheng Wang, Hong-xin Niu, Qian-qian Jia, Xiao-wen Chen, Xiao-yan Du, Lu Lu, Bo Huang, Quan Zhang, Yue Chen, Hai-bo Long

**Affiliations:** ^1^Division of Nephrology, Zhujiang Hospital, Southern Medical University, Guangzhou, Guangdong 510280, China; ^2^Department of Nephrology, Xiangyang Central Hospital, Hubei University of Arts and Science, Xiangyang, Hubei 441021, China; ^3^Department of Nephrology, Zhumadian Central Hospital, Zhumadian, Henan 463000, China; ^4^Department of Traditional Chinese Medicine, Zhujiang Hospital, Southern Medical University, Guangzhou, Guangdong 510280, China; ^5^College of Pharmacy, The State Key Laboratory of Elemento-Organic Chemistry, and Tianjin Key Laboratory of Molecular Drug Research, Nankai University, Tianjin 300071, China

## Abstract

Inflammation is a relevant factor in the pathogenesis of diabetes nephropathy (DN). Sesquiterpene lactones (SLs), originally isolated from *Tanacetum parthenium*, have been reported to exhibit anti-inflammatory effects but few studies have examined their effects on DN. To determine whether advanced oxidation protein products (AOPPs) can induce the expression of chemokine monocyte chemoattractant protein- (MCP-) 1 in cultured mouse podocytes and to explore the mechanisms of the potential renoprotection of SLs, we treated podocytes with AOPPs and SLs (parthenolide and its derivatives micheliolide, compound 1, and compound 2). MCP-1 mRNA and protein expression were tested using quantitative real-time PCR and ELISA, respectively, and the protein levels of IKK*β*, phospho-IKK*β*, I*κ*B*α*, NF-*κ*B p65, phospho-NF-*κ*B p65, and tubulin were analyzed by Western blotting. AOPPs activated the expression of MCP-1 mRNA and protein in a dose- and time-dependent manner, activated IKK*β* and NF-*κ*B p65, and promoted I*κ*B*α* degradation. The IKK/NF-*κ*B inhibitor parthenolide decreased AOPP-induced MCP-1 expression. Pretreatment with SLs inhibited MCP-1 mRNA and protein expression and suppressed IKK*β* and NF-*κ*B p65 phosphorylation and I*κ*B*α* degradation. Taken together, these findings provide a novel explanation for the anti-inflammatory effects of SLs that will ultimately benefit DN and potentially other inflammatory and immune renal diseases.

## 1. Introduction

The latest data from the International Diabetes Federation reveal that there are currently 371 million people living with diabetes, and another 280 million are at high risk of developing the disease. Half of a billion people are expected to be living with diabetes by 2030. DN is a common microvascular complication of diabetes, leading to premature death and end-stage renal disease (ESRD). Remarkably, the excess risk of death from any cause with type 1 or 2 diabetes is associated almost entirely with the presence of kidney disease [1–3]. Recent studies have shown that kidney inflammation is crucial in promoting the development and progression of DN. Inflammation may be a key factor, which is activated by the metabolic, biochemical, and hemodynamic derangements known to exist in the diabetic kidney [4–6]. MCP-1, also known as C-C motif chemokine (CCL) 2, increases progressively in diabetic kidneys in animal models [7]. MCP-1 is involved in the direction of macrophage migration into the diabetic kidney, upregulates the expression of adhesion molecules, and promotes the expression of other proinflammatory cytokines in DN [8]. MCP-1 can be synthesized by mononuclear cells and renal resident cells, including podocytes [5, 9]. The podocyte seems to be a suitable choice for further investigation, as MCP-1 gene expression appears to be predominantly localized to podocytes in the glomeruli of diabetic mice [7]. The latest data show the urinary sediment podocalyxin to creatinine ratio had a positive correlation with the urinary albumin to creatinine ratio and the urinary MCP-1 to creatinine ratio in patients with type 2 diabetes [10]. Other research has shown that once MCP-1 is secreted, it can diffuse back to the podocyte and act in a loop by activating the CCR2 receptor, which then initiates profound biological effects [11], suggesting podocytes as a potential target of inflammation in DN. Considering that podocytes are related to increased proteinuria and contribute to renal progression in DN [12], therapies aimed at preventing or limiting podocyte injury and/or promoting podocyte repair or regeneration may have major clinical and economic benefits [13].

Advanced oxidation protein products (AOPPs) were first discovered and reported as uremic toxins by Witko-Sarsat et al. in 1996 [14] and were found in association with diabetes in later studies [15, 16]. AOPPs are the dityrosine-containing and cross-linking protein products formed during oxidative stress by the reaction of serum protein with chlorinated oxidants and are often carried by albumin in vivo [14, 17]. Recent studies have found that the chronic accumulation of AOPPs promotes inflammation in both the diabetic and nondiabetic kidney by significantly increasing macrophage infiltration and the overexpression of MCP-1 in the remnant kidney and during experimental diabetic nephropathy [18, 19]. AOPPs can also decrease the expression of nephrin and podocin in podocytes, resulting in podocyte apoptosis and deletion [20–22]. Although these studies suggest that the accumulation of AOPPs has an important role in the progression of DN, the mechanism underlying the pathogenic effect of AOPPs remains to be further investigated.

Sesquiterpene lactones, originally isolated from medicinal herbs of the Asteraceae family, have been reported to exhibit a variety of anti-inflammatory, immunomodulatory, and anticancer effects, mostly based on their alkylating capabilities [23, 24]. Parthenolide (PTL) and other SLs have been reported to exhibit anti-inflammatory effects by inhibiting IKK and I*κ*B*α* phosphorylation and/or affecting DNA binding ability [25, 26]. Other studies have demonstrated that PTL blocks MCP-1 mRNA and protein expression by inhibiting NF-*κ*B activity in experimental glomerulonephritis and human renal mesangial cells [27, 28] as well as in podocytes [9, 29].

Recently, we demonstrated that SLs, including PTL, micheliolide (MCL), and several synthetic analogs of these molecules, could effectively attenuate the high glucose-stimulated and AOPP-induced activation of NF-*κ*B, the degradation of I*κ*B*α*, and the expression of MCP-1 in rat mesangial cells (MCs) [30, 31]. These findings showed that SLs can exert a beneficial role in high glucose-stimulated and AOPPs-induced inflammation and ECM deposition by preventing NF-*κ*B activation in MCs. However, whether SLs have anti-inflammatory effects in podocytes under the accumulation of AOPPs has not yet been clarified, and our previous studies were focused on the screening of anti-inflammatory effects of SLs, but the underlying mechanism of action has not yet been elucidated. Thus, the present study aimed to characterize the inflammatory effect of AOPPs in cultured mouse podocytes and, more importantly, we selected MCL, compound 1, and compound 2 according to our previous experiments to further explore the mechanism of the potential anti-inflammatory effects of SLs.

## 2. Materials and Methods

### 2.1. AOPPs-MSA Preparation and Determination

AOPPs-MSA was prepared in vitro according to a previously described method [21]. Briefly, MSA solution (200 mg/mL) was incubated with 200 mM HClO (Fluke, Buchs, Switzerland) at an optimal ratio (MSA to HClO = 1 : 140) for 30 min at 37°C and dialyzed overnight against PBS to remove free HClO. The preparation of AOPPs was passed through a Detoxi-Gel column (Pierce, Rockford, IL, USA) to remove any contaminated endotoxin. Endotoxin levels in the preparation were measured with a Limulus Amoebocyte Lysate kit and were found to be below 0.025 EU/mL. The AOPP content was determined by measuring absorbance at 340 nm in acidic conditions and was calibrated with chloramines-T in the presence of potassium iodide. The content of AOPPs was 72.40 ± 9.8 nmol/mg protein in prepared AOPPs-MSA and 0.2 ± 0.06 nmol/mg protein in native MSA.

### 2.2. Cell Culture of Podocytes and Drugs

**Figure 1:**
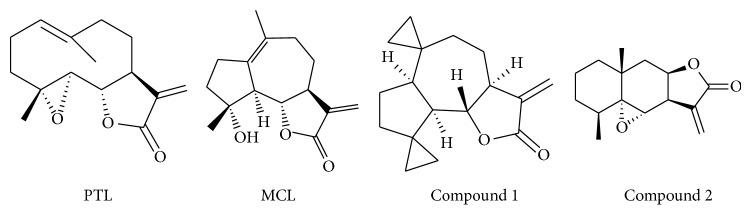
Structures of natural SLs and synthetic derivatives.

Conditionally immortalized mouse podocytes were generously provided by Shankland et al. (Harvard Medical School, Charlestown, MA, USA) and cultured as previously described [32]. Briefly, undifferentiated podocytes were grown in RPMI 1640 containing 10% FBS (BRL Co. Ltd., USA), penicillin (100 U/mL), streptomycin (100 mg/mL), and 50 IU/mL recombinant murine IFN-*γ* (Pepro Tech, USA) at 33°C (permissive conditions). To acquire a differentiated phenotype, podocytes were cultured at 37°C in the absence of IFN-*γ* (nonpermissive conditions) for 10–14 days. Differentiated podocytes were starved in RPMI 1640 for 24 h and then treated with various reagents. Podocytes between passages 10 and 20 were used in all experiments. PTL and MCL were purchased from Accendatech Co., Ltd (Tianjin, China), compound 1 and compound 2 were synthesized following the procedure provided in [33, 34] by Accendatech Co., Ltd (Tianjin, China), and the purity of those four compounds was more than 98% (Figure 1). Depending on the experiment demands, podocytes were divided into different groups and then treated with the drugs or/and AOPPs.

### 2.3. Quantitative Real-Time PCR (qPCR) Analysis

Total RNA from podocytes in all samples was extracted and dissolved in RNA-free water and quantified using UV-clear microplates. Aliquots of each RNA extraction were reverse-transcribed simultaneously into cDNA using M-MLV reverse transcriptase according to the manufacturer's protocol (Invitrogen, USA). The qPCR assay was performed using SYBR Green assays (Applied Biosystems, USA). The thermal cycling conditions comprised a 30-second step at 95°C followed by 40 cycles with denaturation at 95°C for 5 s, annealing at 60°C for 30 s, and extension at 72°C for 60 s. The primers for qPCR, which were designed with Primer Express software, were as follows: MCP-1 (125 bp) forward: 5′-CCCAATGAGTAGGCTGGAGA-3′, reverse: 5′-TCTGGACCCATTCCTTCTTG-3′ and GAPDH (102 bp) forward: 5′-ATTGTCAGCAATGCATCCTG-3′, reverse: 5′-ATGGACTGTGGtcATGAGCC-3′. GAPDH was used as an endogenous reference, and each sample was normalized to its GAPDH content. The results were analyzed using the 2-DDCt method and shown as fold change in expression with respect to the controls (unstimulated cells) for all samples.

### 2.4. Measurements of MCP-1 by ELISA

To quantify the level of MCP-1 protein under various experimental conditions, the level of supernatant MCP-1 was measured using a solid-phase quantitative sandwich enzyme-linked immunosorbent assay (ELISA) kit (eBioscience Inc., USA) for MCP-1 that was specific for mouse MCP-1 and sensitive to 2.2 pg/mL, and the standard curve range was 15.6–1000 pg/mL. The concentration in the culture supernatant was normalized to the total protein content.

### 2.5. Western Blot Analysis

All samples were washed three times with ice-cold PBS and dissolved in RIPA lysis buffer containing 1 mM PMSF for 30 min on ice. The lysates were centrifuged (12,000 ×g) at 4°C for 15 min. The protein concentration was then determined using a BCA protein assay kit. Protein samples (40 *µ*g per well) were separated by 12% SDS-PAGE and transferred onto PVDF membranes (Millipore, Bedford, MA). After being blocked in 5% nonfat milk in Tris-buffered saline (TBS) solution for 1 h, the membranes were incubated overnight at 4°C with the following primary antibodies (Cell Signaling Technologies, Danvers, MA): IKK*β* rabbit mAb (Product #2370), phospho-IKK*α*/*β* rabbit mAb (Product #2697), NF-*κ*B p65 rabbit mAb (Product #8242), phospho-NF-*κ*B p65 rabbit mAb (Product #3033), I*κ*B*α* mouse mAb (Product #4814), and tubulin mouse mAb (Beyotime Institute of Biotechnology, Shanghai, China, Product AT819). Next, the corresponding HRP-conjugated secondary antibodies (Cell Signaling Technologies, Danvers, MA, product #7074, product #7076) were used for 1 h at room temperature. Electrochemiluminescence detection was performed and the protein bands were captured and documented using a CCD system (Image Station 2000 MM, Kodak, Rochester, NY, USA). The intensities of the protein bands were quantified by Molecular Imaging Software Version 4.0, which was provided with the Kodak 2000 MM System. The optical density was normalized against tubulin protein expression levels.

### 2.6. Statistical Analysis

Results are expressed as the means ± SEM and represent assays from at least three independent experiments. Statistical significance was estimated using the one-way analysis of variance (ANOVA). Values with *P* < 0.05 were considered statistically significant. Statistical analyses were performed using SPSS 13.0 (SPSS, Chicago, IL, USA).

## 3. Results

### 3.1. AOPPs Increased the Expression of MCP-1 mRNA and Protein in Cultured Podocytes

**Figure 2:**
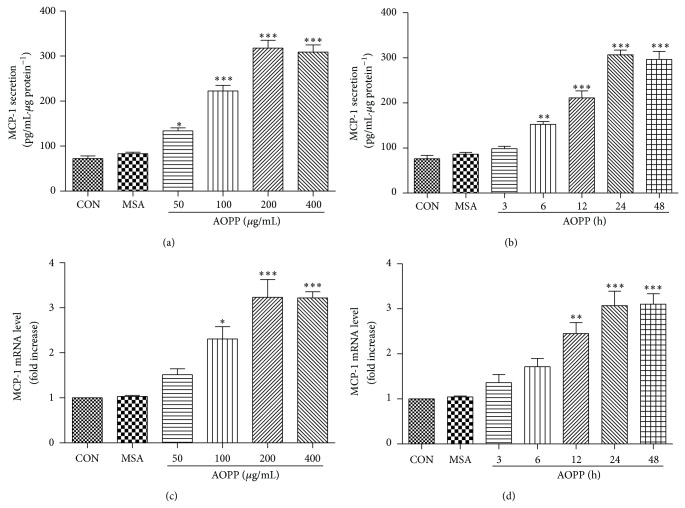
AOPPs stimulation increased the expression of MCP-1 in cultured podocytes. Podocytes were incubated with the indicated concentration of AOPPs for 24 h or 200 *μ*g/mL of AOPPs for the indicated time and subjected to MCP-1 mRNA and protein expression analysis. AOPP treatment increased the expression of MCP-1 at both the mRNA (c, d) and protein levels (a, b) in a dose- and time-dependent manner. Data are expressed as the means ± SD of three independent experiments. ANOVA, ^*^ *P* < 0.05, ^**^ *P* < 0.01, and ^***^ *P* < 0.001 versus CON. CON, untreated cells; MSA, mouse serum albumin.

To investigate whether AOPPs induce the expression of MCP-1 in podocytes, we tested the expression levels of MCP-1 mRNA by qPCR and MCP-1 protein by ELISA. The data showed that AOPPs can promote the expression of MCP-1 protein in a dose-dependent manner and reach a maximum level at a concentration of 200 *μ*g/mL. At the same time, AOPPs can enhance the expression of MCP-1 protein in a time-dependent manner, and a significant increase in MCP-1 protein was observed after a 24 h incubation. qPCR revealed that AOPPs significantly increased the expression of MCP-1 and peaked at 200 *μ*g/mL at 24 h. The expression of MCP-1 was unchanged in cells incubated with native MSA or medium alone, suggesting that the upregulation of MCP-1 was associated with an oxidative modification of MSA (Figure 2).

### 3.2. The Impact of AOPPs on the Expression of IKK*β*, I*κ*B*α*, and NF-*κ*B Protein in Podocytes

**Figure 3:**
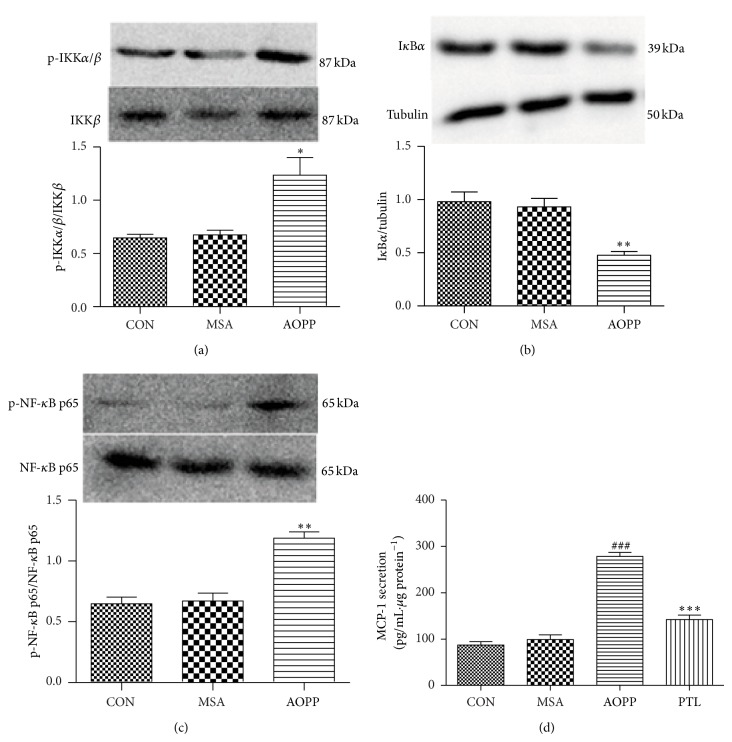
AOPP-increased MCP-1 expression in podocytes was mainly mediated by the IKK/NF-*κ*B pathway. The overnight serum-deprived podocytes were treated with 200 *μ*g/mL AOPPs for 30 min. The expression of p-IKK*α*/*β*, p-NF-*κ*B p65, and I*κ*B*α* was examined by Western blotting (a, b, and c). To verify the role of the IKK/NF-*κ*B pathway involved in the AOPP-induced MCP-1 expression, protein levels of MCP-1 were determined by ELISA using cell supernatants exposed to AOPPs for 24 h in the presence of the IKK/NF-*κ*B inhibitor PTL (d). Data are expressed as the means ± SEM of three independent experiments. ANOVA, (a, b, and c) ^*^ *P* < 0.05, ^**^ *P* < 0.01 versus CON. (d) ^###^ *P* < 0.001 versus CON, ^***^ *P* < 0.001 versus AOPP. CON, untreated cells; MSA, mouse serum albumin.

To examine the involvement of the proinflammatory signaling pathways of IKK/NF-*κ*B in AOPP-induced MCP-1 expression, we first examined the phosphorylation of IKK*β*, degradation of I*κ*B*α*, and activation of NF-*κ*B by Western blotting. As shown above, the levels of phospho-IKK*α*/*β* and phospho-NF-*κ*B p65 protein were upregulated by 200 *μ*g/mL AOPPs (Figures 3(a) and 3(c)), whereas I*κ*B*α* protein expression declined (Figure 3(b)) when compared to unstimulated cells or cells treated with native MSA. Total levels of IKK*β* and NF-*κ*B p65 did not significantly change among each group.

To further confirm the role of activated IKK/NF-*κ*B in the AOPP-induced upregulation of MCP-1, the expression of MCP-1 was measured in podocytes treated with AOPPs in the presence of PTL, an inhibitor of the IKK/NF-*κ*B signaling pathway. As shown in Figure 3(d), PTL significantly decreased the AOPP-induced upregulation of MCP-1.

These data suggest that the AOPP-induced upregulation of MCP-1 was related to the activation of IKK*β* and NF-*κ*B p65 and the degradation of I*κ*B*α*.

### 3.3. The Effect of SLs (MCL, Compound 1, and Compound 2) on the AOPP-Induced Expression of MCP-1 mRNA and Protein

**Figure 4:**
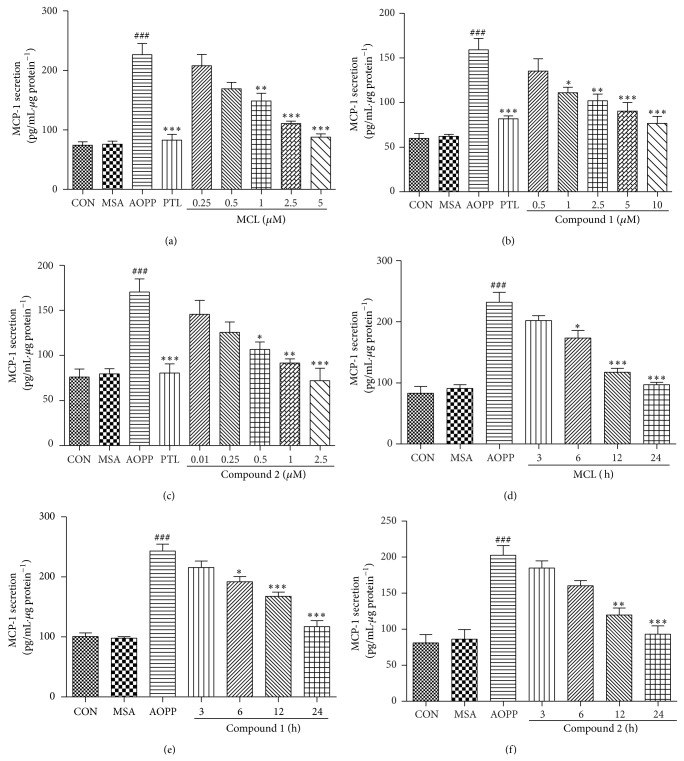
SLs decreased the AOPP-induced expression of MCP-1 protein in a dose- and time-dependent manner. Podocytes were preincubated with the indicated concentration of MCL, compound 1, or compound 2 for 1 h before treatment with 200 *μ*g/mL of AOPPs for 24 h. PTL (5 *μ*M) was used as a positive control (a, b, and c). Aliquots of 200 *μ*g/mL AOPPs were used to treat podocytes for 24 h, and then MCL (5 *μ*M), compound 1 (10 *μ*M), or compound 1 (2.5 *μ*M) was added for the indicated times (d, e, and f). The expression levels of MCP-1 protein were determined by ELISA. SLs decreased the AOPP-induced expression of MCP-1 protein in a dose- and time-dependent manner. The data are expressed as the means ± SEM of three independent experiments. ANOVA, ^###^ *P* < 0.001 versus CON; ^*^ *P* < 0.05, ^**^ *P* < 0.01, and ^***^ *P* < 0.001 versus AOPP. CON, untreated cells; MSA, mouse serum albumin.

**Figure 5:**
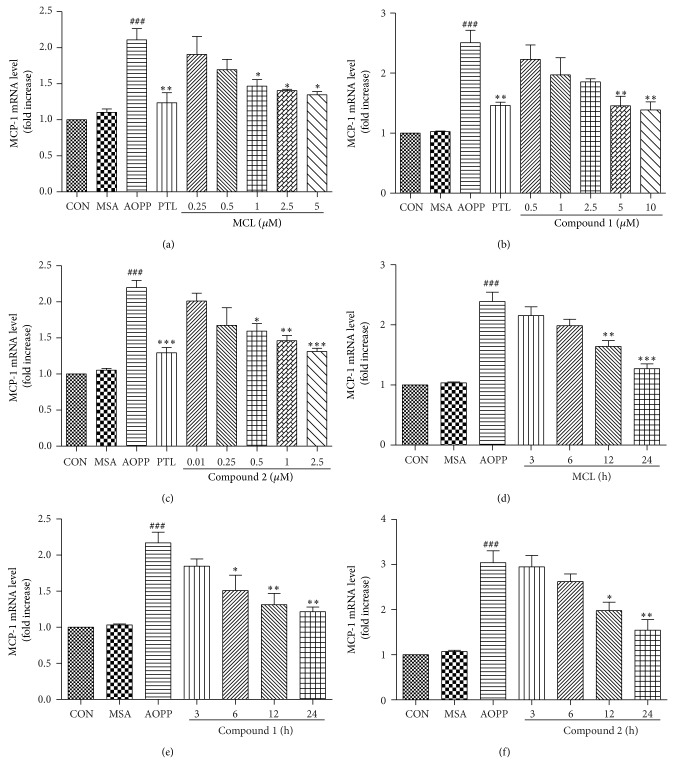
SLs decreased the AOPP-induced expression of MCP-1 mRNA in a dose- and time-dependent manner. Podocytes were preincubated with the indicated concentration of MCL, compound 1, or compound 2 for 1 h before treatment with 200 *μ*g/mL of AOPPs for 24 h. PTL (5 *μ*M) was used as a positive control (a, b, and c). Aliquots of 200 *μ*g/mL AOPPs were used to treat podocytes for 24 h, and then MCL (5 *μ*M), compound 1 (10 *μ*M), or compound 1 (2.5 *μ*M) was added for the indicated times (d, e, and f). The expression levels of MCP-1 mRNA were determined by qPCR. SLs decreased the AOPP-induced expression of MCP-1 mRNA in a dose- and time-dependent manner. The data are expressed as the means ± SEM of three independent experiments. ANOVA, ^###^ *P* < 0.001 versus CON; ^*^ *P* < 0.05, ^**^ *P* < 0.01, and ^***^ *P* < 0.001 versus AOPP. CON, untreated cells; MSA, mouse serum albumin.

Next, we tested the hypothesis that SLs (MCL, compound 1, and compound 2), as derivatives of PTL, may have the same anti-inflammatory response in podocytes. After preincubation with SLs, cells were treated with 200 *μ*g/mL AOPPs for 24 h. PTL (5 *μ*M) was used as a positive control. The expression of MCP-1 mRNA was determined by qPCR, and MCP-1 protein expression was measured by ELISA. The results presented in Figures 4(a), 4(b), and 4(c) and Figures 5(a), 5(b), and 5(c) demonstrated that MCL, compound 1, and compound 2 responded in a dose-dependent manner to prevent the expression of MCP-1 mRNA and protein. Additionally, MCL, compound 1, and compound 2 at a concentration of 5 *μ*M, 10 *μ*M, and 2.5 *μ*M, respectively, produced an almost equal inhibitory effect on MCP-1 as 5 *μ*M of PTL.

To further examine the effect of treatment time when using SLs, 200 *μ*g/mL of AOPPs was used to treat podocytes for 24 h along with the addition of SLs for the indicated time. As shown in Figures 4(d), 4(e), and 4(f) and Figures 5(d), 5(e), and 5(f), MCL, compound 1, and compound 2 treatment resulted in a time-dependent prevention of MCP-1 mRNA and protein expression, and a longer exposure time of SLs during a 24 h period showed a greater inhibition of MCP-1 expression.

Our data reveal that these three compounds have an activity profile similar to that of PTL. MCL, compound 1, and compound 2 could inhibit the expression of MCP-1 mRNA and protein induced by AOPPs in a dose- and time-dependent manner in podocytes.

### 3.4. The Effects of SLs (MCL, Compound 1, and Compound 2) on the Activation of IKK*β* and NF-*κ*B and the Degradation of I*κ*B*α* by AOPPs

**Figure 6:**
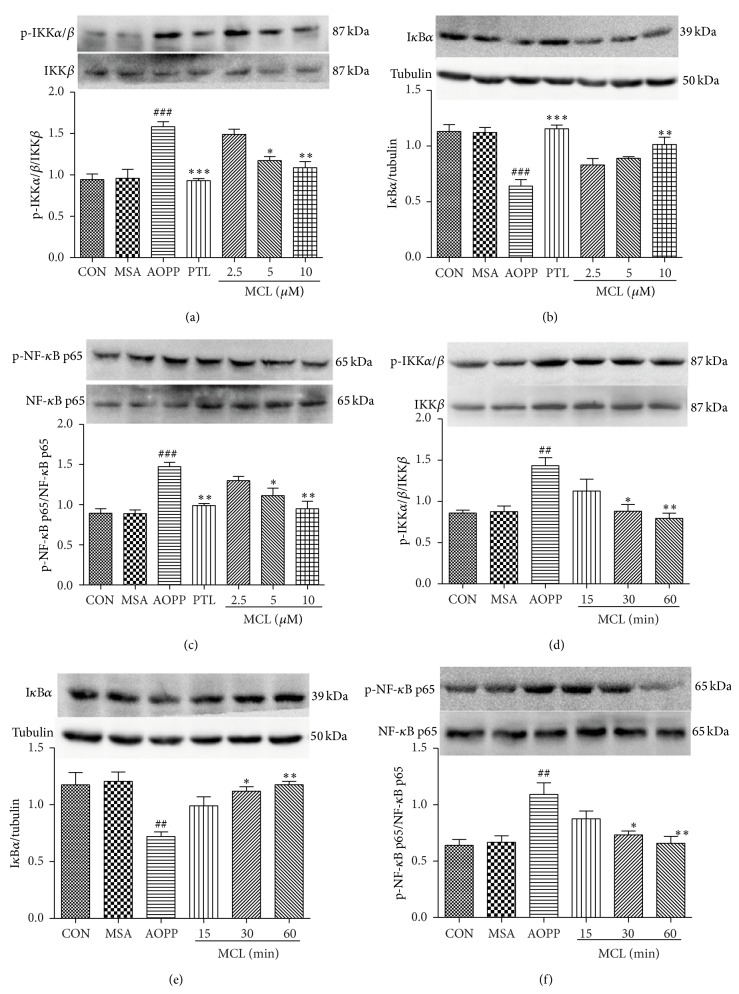
MCL prevented AOPP-induced IKK*β* and NF-*κ*B p65 activation and I*κ*B*α* degradation in a dose- and time-dependent manner in podocytes. After 24 h of serum starvation, podocytes were pretreated with MCL (2.5 *μ*M, 5 *μ*M, and 10 *μ*M) for 1 h and then exposed to 200 *μ*g/mL AOPPs or native MSA for 30 min. PTL (10 *μ*M) was used as a positive control (a, b, and c). Alternatively, podocytes were treated with 200 *μ*g/mL AOPPs for 30 min, and then MCL (10 *μ*M) was added for a preset time (d, e, and f). The protein expression of p-IKK*α*/*β*, p-NF-*κ*B p65, and I*κ*B*α* was analyzed by Western blotting. The data are expressed as the means ± SEM of three independent experiments. ANOVA, ^##^ *P* < 0.01, ^###^ *P* < 0.001 versus CON; ^*^ *P* < 0.05, ^**^ *P* < 0.01, and ^***^ *P* < 0.001 versus AOPP. CON, untreated cells; MSA, mouse serum albumin.

**Figure 7:**
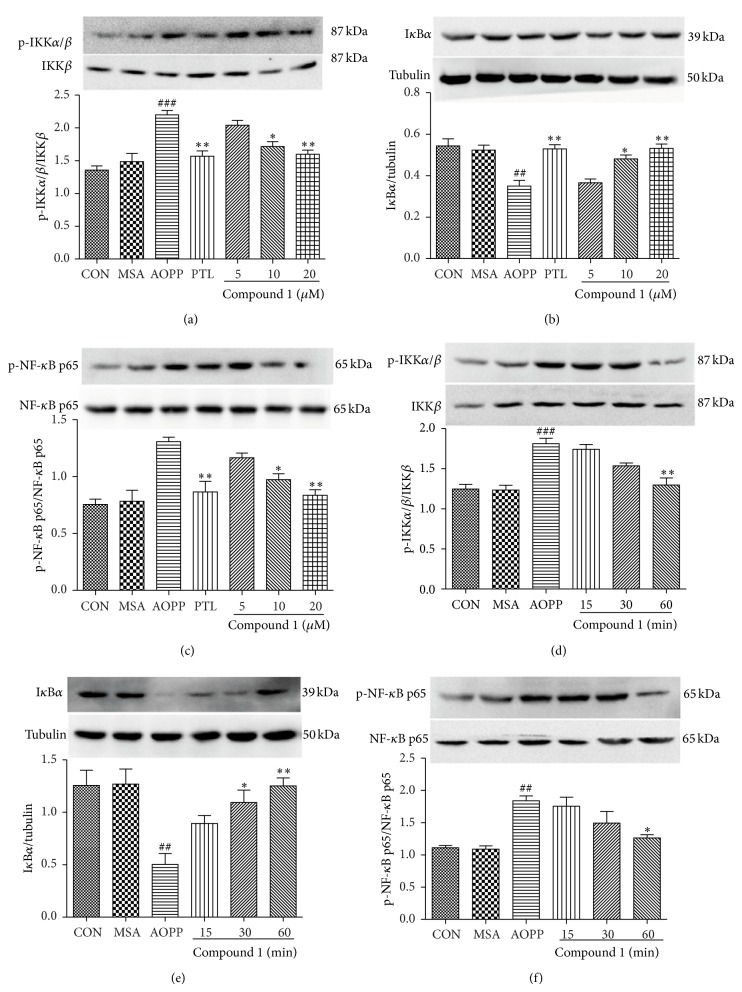
Compound 1 prevented AOPP-induced IKK*β* and NF-*κ*B p65 activation and I*κ*B*α* degradation in a dose- and time-dependent manner in podocytes. After 24 h of serum starvation, podocytes were pretreated with compound 1 (5 *μ*M, 10 *μ*M, and 20 *μ*M) for 1 h and then exposed to 200 *μ*g/mL AOPPs or native MSA for 30 min. PTL (10 *μ*M) was used as a positive control (a, b, and c). Alternatively, podocytes were treated with 200 *μ*g/mL AOPPs for 30 min, and then compound 1 (20 *μ*M) was added for a preset time (d, e, f). The protein expression of p-IKK*α*/*β*, p-NF-*κ*B p65, and I*κ*B*α* was analyzed by Western blotting. The data are expressed as the means ± SEM of three independent experiments. ANOVA, ^##^ *P* < 0.01, ^###^ *P* < 0.001 versus CON; ^*^ *P* < 0.05, ^**^ *P* < 0.01 versus AOPP. CON, untreated cells; MSA, mouse serum albumin.

**Figure 8:**
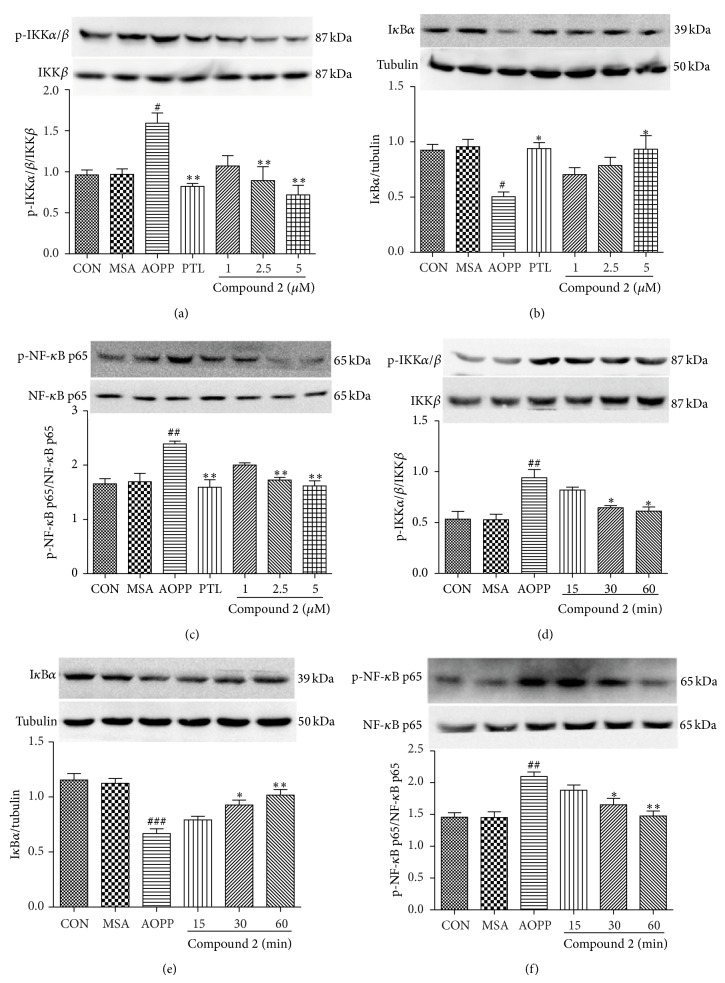
Compound 2 prevented AOPP-induced IKK*β* and NF-*κ*B p65 activation and I*κ*B*α* degradation in a dose- and time-dependent manner in podocytes. After 24 h of serum starvation, podocytes were pretreated with compound 2 (1 *μ*M, 2.5 *μ*M, and 5 *μ*M) for 1 h and then exposed to 200 *μ*g/mL AOPPs or native MSA for 30 min. PTL (10 *μ*M) was used as a positive control (a, b, and c). Alternatively, podocytes were treated with 200 *μ*g/mL AOPPs for 30 min, and then compound 2 (5 *μ*M) was added for a preset time (d, e, and f). The protein expression of p-IKK*α*/*β*, p-NF-*κ*B p65, and I*κ*B*α* was analyzed by Western blotting. The data are expressed as the means ± SEM of three independent experiments. ANOVA, ^#^ *P* < 0.05, ^##^ *P* < 0.01, and ^###^ *P* < 0.001 versus CON; ^*^ *P* < 0.05, ^**^ *P* < 0.01 versus AOPP. CON, untreated cells; MSA, mouse serum albumin.

To further analyze whether factors of the NF-*κ*B activation cascade are influenced by SLs, we pretreated podocytes with different concentrations of MCL, compound 1, and compound 2 for 1 h before incubation with 200 *μ*g/mL of AOPPs or native MSA for 30 min or 200 *μ*g/mL AOPPs for 30 min, and we also added SLs for the indicated times. As shown in Figures 6(a), 6(b), and 6(c), Figures 7(a), 7(b), and 7(c), and Figures 8(a), 8(b), and 8(c), MCL, compound 1, and compound 2 prevented the protein expression of phospho-IKK*α*/*β* and phospho-NF-*κ*B p65 and increased the protein expression of I*κ*B*α* in a dose-dependent manner. The effect of MCL, compound 1, and compound 2 on inhibiting the activation of IKK*β*, degradation of I*κ*B*α*, and activation of NF-*κ*B peaked at 10 *μ*M, 20 *μ*M, and 5 *μ*M, respectively, and almost reached the effect of 10 *μ*M of PTL. Figures 6(d), 6(e), and 6(f), Figures 7(d), 7(e), and 7(f), and Figures 8(d), 8(e), and 8(f) suggest that these three compounds prevented the phosphorylation of IKK*β* and NF-*κ*B p65 and the degradation of I*κ*B*α* protein in a time-dependent manner.

## 4. Discussion

Our data clearly showed that AOPPs upregulated the mRNA and protein expression of MCP-1 and activated proteins closely related to the NF-*κ*B signaling pathway. PTL, as a potent inhibitor of NF-*κ*B, prevented this effect of AOPPs. The application of SLs (MCL, compound 1, and compound 2) in podocytes downregulated AOPP-induced MCP-1 expression and inhibited the proteins closely associated with the NF-*κ*B signaling pathway. In summary, the findings indicate that AOPPs stimulate the expression of the chemokine MCP-1 through an IKK/NF-*κ*B-dependent signaling pathway in cultured differentiated mouse podocytes. More importantly, we have demonstrated that SLs and their derivatives were able to significantly decrease AOPP-induced MCP-1 expression in podocytes by inhibiting the IKK/NF-*κ*B pathway, suggesting that SLs may protect against DN as well as other inflammatory and immune renal diseases mainly through NF-*κ*B inhibition and anti-inflammatory effects.

Recent studies on AOPPs, as a class of potential renal pathogenic mediators, have highlighted the importance of determining the mechanisms by which AOPPs might induce or promote the progression of glomerulopathy. According to previous studies, there are several major pathogenic mechanisms for AOPPs: (1) Central link: the PKC-NADPH oxidase-dependent activation of ROS [21, 22, 35, 36]; (2) an AOPPs-RAGE interaction [22, 37]; and (3) a CD36-dependent pathway involving the activation of NF-*κ*B/AP-1 [36, 38]. However, the role of IKK/NF-*κ*B has not yet been clearly illuminated. Increasing evidence supports the notion that the IKK/NF-*κ*B pathway plays a role in the induction and maintenance of the state of inflammation that underlies metabolic diseases such as obesity and type 2 diabetes [39, 40]. NF-*κ*B normally resides in the cytosol in an inactive complex with an I*κ*B family member, such as I*κ*B*α*, and this interaction prevents NF-*κ*B from entering the nucleus and activating DNA transcription. However, once the IKK complex is activated, the IKK phosphorylation of I*κ*B molecules promotes their degradation and the release of NF-*κ*B, which then translocates to the nucleus to promote the transcription of target genes. IKK*β*, as a subunit of the IKK complex, has been shown to be an essential mediator of the inflammatory process [39–41]. Researchers have reported that AOPPs could activate NF-*κ*B, which plays a critical role in the regulation of inflammatory cytokines [36, 42]. We found that the accumulation of AOPPs increased NF-*κ*B p65 phosphorylation. Notably, we also demonstrated that AOPPs activated IKK*β* and I*κ*B*α* in cultured podocytes. Consistent with this observation, our previous study demonstrated that the administration of AOPPs induced a similar reaction in cultured rat MCs [31].

Enough evidence has accumulated to prove a close relationship between inflammation and DN [4, 43, 44]. MCP-1 expression is significantly increased in DN, and macrophage infiltration into the glomeruli is associated with glomerular injury. MCP-1-null mice are protected against DN [33]. In the present study, we found that AOPPs significantly increased MCP-1 expression in podocytes. In addition, our results demonstrate that AOPP-induced MCP-1 expression is mainly mediated by the IKK/NF-*κ*B-dependent signaling pathway because PTL, a potent inhibitor of NF-*κ*B, decreased this AOPP-induced MCP-1 expression.

PTL have been proved to gradually show its anti-inflammatory effect [25, 26]. Studies have shown that PTL blocks the expression of MCP-1 mRNA and protein through the inhibition of IKK activity, thereby preventing I*κ*B degradation and inhibiting NF-*κ*B translocation [27, 28]. In our previous studies, we found that PTL could inhibit high glucose-induced and AOPP-induced NF-*κ*B activation and MCP-1 expression in MCs [30, 31]. Here, we found that PTL could suppress the MCP-1 expression upregulated by AOPPs via the IKK/NF-*κ*B pathway. Notably, other studies have demonstrated that PTL derivatives have similar anticancerous effects as PTL; moreover, the water-soluble form of MCL, DMAMCL, demonstrated superior efficacy when compared to DMAPT in the treatment of an acute leukemia mouse model [34, 45]. Similarly, our previous studies indicated that certain analogs of PTL have distinctly anti-inflammatory effects [30, 31]. The data from the present study show that MCL, compound 1, and compound 2, as PTL analogs, exhibit varying degrees of anti-inflammatory effects through the inhibition of the IKK/NF-*κ*B pathway. Interestingly, in addition to the similar bioactivities between PTL and the synthetic compounds, the synthetic compounds were substantially more stable and presented fewer side effects compared to PTL. Therefore, the synthetic SL derivatives may have a broad scope in future clinical applications with regard to inflammatory and immune renal diseases, including DN.

In summary, the accumulation of AOPPs promotes an inflammatory response in podocytes, and this response is mainly mediated by IKK/NF-*κ*B activation. Remarkably, we found that SLs are able to inhibit AOPP-induced MCP-1 expression in podocytes, and the salutary effects of SLs are likely mediated by their anti-inflammatory properties through the inhibition of the IKK/NF-*κ*B pathway. As a major complication of diabetes, DN often leads to ESRD and high mortality, and finding an effective treatment and prevention for DN has been a major challenge facing modern medicine. The observations presented in this study demonstrate the potential renoprotective utility of SLs and suggest a therapeutic agent for the treatment of inflammation in DN and potentially other inflammatory and immune renal diseases.
